# Evaluation of the Possible Protective Role of Nobiletin against Arsenic-Induced Liver Damage in Male Albino Rats

**DOI:** 10.3390/toxics11020110

**Published:** 2023-01-24

**Authors:** Muhammad Umar Ijaz, Aqsa Ahmed, Khalid Abdullah Al-Ghanim, Fahad Al-Misned, Mian Nadeem Riaz, Zahid Ali Kaimkhani, Shahid Mahboob

**Affiliations:** 1 Department of Zoology, Wildlife and Fisheries, University of Agriculture, Faisalabad 38040, Pakistan; 2 Department of Zoology, College of Science, King Saud University, P.O. Box 2455, Riyadh 11451, Saudi Arabia; 3 Texas A&M University, College Station, TX 2476, USA; 4 College of Medicine, King Saud University, Riyadh 11451, Saudi Arabia

**Keywords:** arsenic, reactive oxygen species, inflammation, apoptosis, nobiletin, antioxidant

## Abstract

Arsenic (As) is a toxic contaminant present in organic and inorganic forms in the environment. Nobiletin (NOB) is a polymethoxy flavone that has recently gained substantial consideration due to its curative impacts. The present experiment was conducted to assess the hepatoprotective efficiency of NOB on As-generated hepatotoxicity. Twenty-four adult rats were equally distributed into four groups and designated as control, As (50 mg/kg)-treated, As + NOB (50 mg/kg and 25 mg/kg, respectively), and NOB (25 mg/kg)-treated groups. After 30 days, experimental animals were decapitated, then blood and tissue samples were collected for further analysis. The group treated with As showed a significant decrease in the activity of antioxidant enzymes, including catalase (CAT), superoxide dismutase (SOD), peroxidase (POD), glutathione (GSH), glutathione reductase (GSR), and total antioxidant status (TAS), and a substantial increase in the accumulation of As in liver tissues, levels of total oxidant status (TOS), hydrogen peroxide (H_2_O_2_), and lipid peroxidation (TBARS). Significant increases in alanine aminotransferase (ALT), alkaline phosphatase (ALP), and aspartate aminotransferase (AST) levels were observed in As-treated rats. Moreover, nuclear factor (NF)-κB, tumor necrosis factor (TNF)-α, interleukin (IL)-1β, interleukin (IL)-6, and cyclo-oxygenase (COX)-2 activity, as well as the levels of pro-apoptotic markers (Bax, Caspase-3, and Caspase-9) were increased on exposure to As. In contrast, the anti-apoptotic marker (Bcl-2) level was significantly decreased. As administration showed a significant disturbance in hepatic tissue histology. However, cotreatment of NOB with As considerably increased the antioxidant enzyme activity, with a noteworthy reduction in the deposition of As in hepatic tissues, TBARS, and H_2_O_2_ levels. NOB-administrated rats showed considerable recovery in terms of inflammation, apoptosis, and histological damage. Hence, NOB can be considered a useful curative compound due to its medicinal properties against As-prompted hepatotoxicity.

## 1. Introduction

A sulfhydryl-reactive metalloid, arsenic (As), is among the most harmful naturally occurring contaminants in the environment, universally present in organic and inorganic forms [1]. The primary routes for exposure to As are contaminated food, air, and water [2]. As is recognized as a human carcinogen that globally harms millions [3]. Epidemiological studies have revealed that prolonged exposure to inorganic forms of As is directly related to biochemical and physiological impairments in the liver. Chronic toxicity prompted by As is of tremendous concern as it is associated with bladder, skin, kidney, and liver cancer [4]. However, the actual mechanism behind its carcinogenicity has yet to be thoroughly understood. However, various studies have confirmed that As shows an affinity for the SH-group of proteins, and it hinders the actions of free radical scavengers and raises the production of reactive oxygen species (ROS) (H_2_O_2_ and Superoxide), which causes lipid peroxidation [5]. Accumulation of ROS can lead to several pathological injuries [6] and promotes oxidative disruption in the cell [7].

The liver is considered an ideal place for As-induced lethal effects, as it performs a pivotal role in the metabolism and detoxification of toxins [8]. A practical method must be executed to overcome this predicament and enhance the antioxidant-defense mechanism by using certain antioxidants to bring down the As-generated oxidative stress because, at the moment, oxidative stress is a well-pronounced mechanism behind As-induced liver toxicity [9].

Flavonoids have attracted much attention during the last few years [7]. Nobiletin (NOB) is a polymethoxy-flavone extracted from a variety of citrus fruits, which has attracted much attention due to its several health-promoting effects, such as antioxidant [10], anti-inflammatory [11], anti-diabetic, and anti-cancer [12]. Furthermore, numerous investigations have indicated that NOB plays a defensive role in the nervous system, kidney, and heart [13]. The potential mitigative effects of NOB on the oxidative stress caused by As are not fully understood. 

This research work assessed the hepatoprotective and antioxidant efficiency of NOB against As-prompted hepatotoxicity and oxidative stress in rat liver by assessing the As accumulation, antioxidant enzymes, total oxidant/antioxidant status, lipid peroxidation, liver functioning enzymes level, inflammation, apoptosis, and histopathology.

## 2. Materials and Methods

### 2.1. Chemicals

Both NOB and As were bought from Sigma-Aldrich (Darmstadt, Germany). In comparison, other reagents utilized in this experiment were of analytical grade quality and were purchased from standard manufacturers.

### 2.2. Animals

Sprague Dawley rats (n = 24) weighing (220–250 g) were taken from the experimental animal unit of the University of Agriculture, Faisalabad. Animals were kept in metal cages (six rats/cage) and provided with a controlled laboratory environment. Prior to starting the experiment, the rats were adjusted to their surroundings for 7 days. Animals were handled and treated in compliance with the guidelines prepared by the Institutional Ethics Committee, University of Agriculture, Faisalabad.

### 2.3. Experimental Design

After one week of acclimatization, the rats were distributed into four groups of six rats each. Rats in group I (Control group) received a standard diet and tap water. Group II rats were orally provided with As at a dose of 50 mg/kg in tap water until the end of the experiment. Animals in group III were orally treated with 50 mg/kg As in tap water and 25 mg/kg of NOB during the investigation. Group IV animals were treated with an oral dose of NOB (25 mg/kg) throughout the trial. The 25 mg/kg dose of NOB was used in accordance with Ijaz et al [14]. After 30 days of treatment, the rats in each group were anesthetized by diethyl ether and killed by decapitation. Blood samples were taken in heparin-holding tubes. Plasma was separated via centrifugation at 3000 rpm for 10 min and stored at −20 °C for further assays. The liver was immediately removed, washed in normal saline, and sliced into two equal pieces. For histological observation, one slice was fixed in a 10% formalin buffer solution. In contrast, the second slice of the liver was homogenized in chilled phosphate buffer saline, centrifuged at 13,000 rpm for 15 min, and the resulting supernatant was set apart, stored at −20 °C, and used for further assessment.

### 2.4. Assessment of As level

The arsenic level was detected through atomic-absorption spectrometry (Perkin Elmer, AAnalyst 400, Waltham, MA, USA) by Brook et al. [15].

### 2.5. Biochemical Analysis

Catalase (CAT) and peroxidase (POD) activities were estimated using a method outlined by Chance and Maehly [16]. However, superoxide dismutase (SOD) activity was evaluated by following the protocol explained by Kakkar et al. [17]. The technique of Carlberg and Mannervik [18] was used to estimate glutathione reductase (GSR) activity. Glutathione (GSH) content in the hepatic tissues homogenates was assessed by following the methodology of Jollow et al. [19]. TOS and TAS were evaluated with the kits (Rel Assay Diagnosing kit; Mega-Tip, Gaziantep, Turkey) used by Erel [20,21]. TBARS levels in liver tissues were evaluated following the protocol illustrated by Iqbal et al. [22]. For the evaluation of H_2_O_2_ levels in tissue homogenates, the methodology used by Pick and Keisari [23] was followed. The total liver protein content was measured using the protocol used by Lowry [24].

### 2.6. Assessment of Liver Function Markers

The evaluation of hepatic (ALT, AST, and ALP) enzymes was conducted using the standardized methodology available on diagnostic kits provided by Wiesbaden (Wiesbaden, Germany).

### 2.7. Inflammatory Markers

NF-κB, TNF-α, IL-1β, and IL-6 levels and COX-2 activity were assessed with standardized diagnostic kits (ELISA) as per the manufacturer’s guidance, BioTek (Winooski, VT, USA).

### 2.8. Apoptosis

Bcl-2, Bax, Caspase-3, and Caspase-9 levels were evaluated with ELISA kits acquired from Cusabio Technology Llc (Houston, TX, USA).

### 2.9. Histomorphometry

Liver samples were embedded in a fixative solution containing 10% formalin, 70% alcohol, and 10% glacial acetic acid, dehydrated in ethanol, and then set in paraffin wax. The tissue samples were sliced into thin sections of 4–5 μm by microtome and stained with hematoxylin/eosin. Then, morphological impairments were studied using a light microscope at 40× magnification to investigate the impact of As and NOB.

### 2.10. Statistical Analysis

All values obtained from the experiment were shown as Mean ± SEM. The data obtained for the differences between the different groups were evaluated by applying one-way analysis of variance (ANOVA) using graph-pad prism software. The means were compared by using Tukey’s test, and the level of significance was adjusted at *p* < 0.05.

## 3. Results

### 3.1. Defensive Role of NOB on Endogenous Antioxidant Profile

The activities of antioxidant enzymes were determined to check the redox potential in hepatic tissues. Fluctuations in antioxidant profiles due to As toxicity were evaluated by assessing the activity of antioxidant enzymes such as CAT, POD, SOD, GSH, and GSR, as shown in (Table 1). The activity of these free radical scavengers was significantly reduced (*p* < 0.05) after As administration compared to the control group. In contrast, cotreatment with NOB effectively attenuated the As poisoning and markedly (*p* < 0.05) improved the activity of protective antioxidants.

Table 2 indicates that As toxicity was associated with augmented LPO, evidenced by a significant elevation in TBARS and H_2_O_2_ concentrations. The findings of this experiment showed that As exposure led to a remarkable (*p* < 0.05) increase in TOS, TBARS, and H_2_O_2_ levels, and a considerable decrease in TAS level compared to the control group. However, co-exposure of NOB significantly (*p* < 0.05) averted the elevation in TBARS, H_2_O_2_, and TOS levels, while increasing the TAS level compared to the As group.

### 3.2. Defensive Role of NOB on Liver Function Indices

The level of hepatic enzymes (ALT, AST, and ALP) was assessed to demonstrate liver injuries. A significant (*p* < 0.05) reduction in the concentration of hepatic enzymes (ALT, AST, and ALP) in the As-treated rats was recorded compared to the control group (Table 3). It was observed that the cotreatment of NOB + As caused a significant (*p* < 0.05) hepatoprotective effect and augmented the level of hepatic enzymes compared to As alone treated rats.

### 3.3. Effect of NOB on Inflammatory Markers

The levels of inflammatory markers were estimated to check the inflammatory profile. As exposure considerably (*p* < 0.05) escalated the levels of NF-κB, TNF-α, IL-1β, IL-6, and COX-2 activity in the As-treated group versus the control group. There was a significant (*p* < 0.05) decrease in the levels and activity of all the inflammatory markers compared to the As group compared to NOB + As co-treatment (Table 4).

### 3.4. Effect of NOB on Pro-Apoptotic and Anti-Apoptotic Markers

The level of apoptotic and anti-apoptotic markers was determined to assess apoptotic and antiapoptotic profiles. In this investigation, As-generated damage in hepatic tissues included an elevated level of Caspase-3, Bax, and Caspase-9. Nonetheless, the level of Bcl-2 was (*p* < 0.05) alleviated in contrast to the control group. Co-treatment of NOB reduced the toxic impacts of As and effectively returned the levels of Bcl-2, Caspase-3, Bax, and Caspase-9 to close to the control group compared with the As-induced group (Table 5).

### 3.5. Defensive Role of NOB on Hepatic Structural Viability

**Figure 1:**
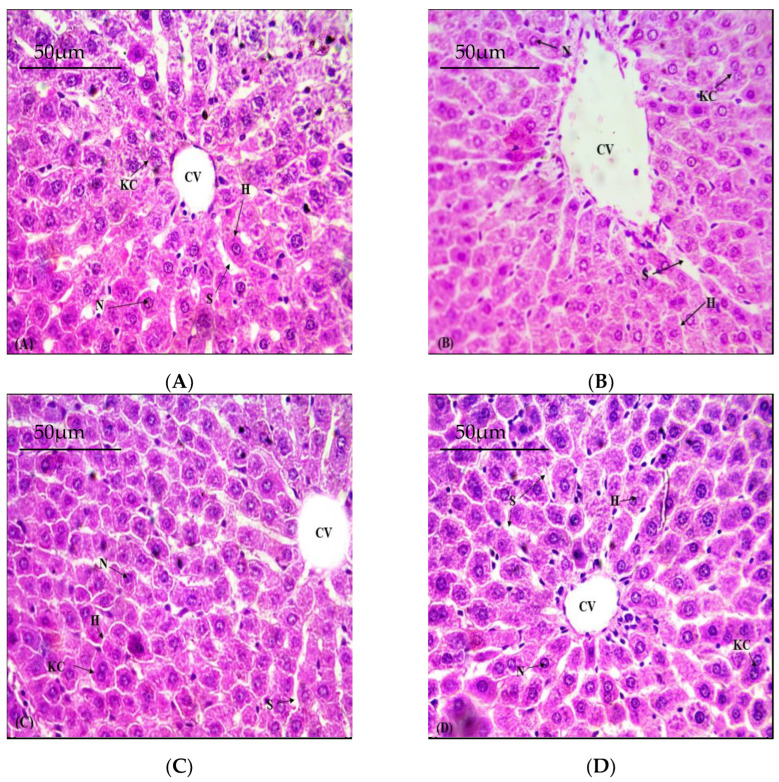
Photomicroscopy of hepatic tissues of all groups (H&E 40X). (**A**) Control group; displaying normal histology. (**B**) As (50 mg/kg); tissues show remarkable necrosis and deterioration. (**C**) As (50 mg/kg) + NOB (25 mg/kg); show reduction in necrosis in the liver tissues followed by improvement in damaged hepatic tissues. (**D**) Supplementation with NOB (25 mg/kg); normal histology almost as in the control group. KC; Kupffer cells. CV; Central venule. S; Sinusoids. N; Nucleus. H; Hepatocytes.

As inebriation had lethal effects on the structural integrity of the liver, as shown in (Figure 1). Liver tissues of control group rats exhibited normal cellular morphology with a regular cord-like arrangement of hepatocytes (Figure 1A). However, treatment with As resulted in severe distortion of cellular morphology with apparent congestion of the portal vein, a more dilated sinusoid, and an irregular arrangement of liver cells (Figure 1B). However, significant attenuation in the intensity of blood vessel congestion, inflammation, necrosis, as well as sinusoidal dilation, were observed via cotreatment with NOB (Figure 1C) when compared to the As group. Thus, co-administration with NOB proved to be helpful in the recovery of the morphology of hepatic tissues.

### 3.6. Effects of NOB on As Accumulation in Hepatic Tissues

The treated group showed an increase in As in the hepatic tissues of rats (*p* < 0.05) compared with the control group. Nonetheless, As levels in the co-treated group (NOB + As) were considerably (*p* < 0.05) reduced compared to the As group. Nevertheless, the NOB-supplemented group showed the same results as the control group.

**Table 1:** Mean ± SEM of As bioaccumulation in liver and effects of As and NOB on the antioxidant enzymes (CAT, SOD, POD, GSH, and GSR).

Group	As Level (µg/gm Tissue)	CAT (U/mg Protein)	SOD (U/mg Protein)	POD (Nanomole)	GSH (μM/g Tissue)	GSR (nm NADPH Oxidized/min/mg Tissue)
Control	1.43 ± 0.10 ^a^	7.36 ± 0.33 ^a^	5.36 ± 0.12 ^a^	7.16 ± 0.21 ^a^	17.54 ± 0.37 ^a^	3.80 ± 0.04 ^a^
As (50 mg/kg)	30.8 ± 2.29 ^b^	4.25 ± 0.36 ^b^	3.03 ± 0.04 ^b^	3.07 ± 0.13 ^b^	6.766 ± 0.40 ^b^	2.02 ± 0.09 ^b^
As (50 mg/kg) + NOB (25 mg/kg each)	6.32 ± 0.35 ^c^	7.21 ± 0.23 ^a^	4.56 ± 0.07 ^c^	6.52 ± 0.15 ^c^	14.23 ± 0.28 ^c^	3.06 ± 0.06 ^c^
NOB (25 mg/kg)	0.85 ± 0.04 ^a^	7.33 ± 0.26 ^a^	4.77 ± 0.06 ^c^	7.05 ± 0.17 ^a^	17.43 ± 0.51 ^a^	3.48 ± 0.07 ^ac^

Values having different superscripts in the same column are significantly different from other groups.

**Table 2:** Mean ± SEM of As and NOB effects on the TAS, TOS, TBARS, and H_2_O_2_ levels.

Group	TAS (mmol/L)	TOS (µmol/L)	TBARS (nm TBARS/min/mg Tissue)	H_2_O_2_ (nM/min/mg Protein)
Control	2.99 ± 0.06 ^a^	8.99 ± 0.23 ^a^	14.29 ± 0.68 ^a^	1.45 ± 0.05 ^a^
As (50 mg/kg)	1.47 ± 0.14 ^b^	14.3 ± 0.38 ^b^	25.14 ± 1.09 ^b^	6.04 ± 0.09 ^b^
As (50 mg/kg) + NOB (25 mg/kg each)	2.53 ± 0.12 ^a^	10.3 ± 0.25 ^c^	17.54 ± 0.19 ^c^	2.38 ± 0.08 ^c^
NOB (25 mg/kg)	2.96 ± 0.11 ^a^	9.21 ± 0.39 ^a^	14.84 ± 0.36 ^a^	1.78 ± 0.07 ^a^

Values having different superscripts in the same column are significantly different from other groups.

**Table 3:** Effects of As and NOB on ALT, AST, and ALP. Values are expressed as Mean ± SEM.

Group	ALT (U/l)	AST (U/l)	ALP (U/l)
Control	37.93 ± 6.37 ^a^	50.55 ± 2.87 ^a^	77.81 ± 2.94 ^a^
As (50 mg/kg)	131.4 ± 5.47 ^b^	146.2 ± 37.6 ^b^	144.1 ± 3.21 ^b^
As (50 mg/kg) + NOB (25 mg/kg each)	65.03 ± 3.21 ^c^	66.51 ± 3.54 ^c^	94.59 ± 4.06 ^c^
NOB (25 mg/kg)	38.25 ± 7.03 ^a^	46.63 ± 3.66 ^d^	79.21 ± 3.09 ^a^

Values having different superscripts in the same column are significantly different from other groups.

**Table 4:** Mean ± SEM of As and NOB effects on the inflammatory markers.

Group	NF-κB (ng/g Tissue)	TNF-α (ng/g Tissue)	IL-1β (ng/g Tissue)	IL-6 (ng/g Tissue)	COX-2 (ng/g Tissue)
Control	12.25 ± 0.36 ^a^	7.12 ± 0.13 ^a^	21.39 ± 0.87 ^a^	7.01 ± 0.22 ^a^	17.56 ± 0.69 ^a^
As (50 mg/kg)	65.99 ± 1.90 ^b^	17.28 ± 0.36 ^b^	87.40 ± 1.30 ^b^	33.16 ± 0.80 ^b^	71.84 ± 1.42 ^b^
As (50 mg/kg) + NOB (25 mg/kg each)	23.62 ±1.34 ^c^	8.98 ± 0.35 ^c^	33.44 ± 1.28 ^c^	18.79 ± 0.64 ^c^	27.35 ± 1.47 ^c^
NOB (25 mg/kg)	12.08 ± 0.43 ^a^	7.09 ± 0.13 ^a^	21.15 ± 0.84 ^a^	6.97 ± 0.23 ^c^	17.51 ± 0.69 ^a^

Values having different superscripts in the same column are significantly different from other groups.

**Table 5:** Mean ± SEM of As and NOB effects on pro-apoptotic and anti-apoptotic markers.

Group	Bcl-2	Bax	Caspase-3	Caspase-9
Control	15.85 ± 0.57 ^a^	2.96 ± 0.11 ^a^	1.64 ± 0.04 ^a^	3.31 ± 0.11 ^a^
As (50 mg/kg)	3.5 ± 0.41 ^b^	9.43 ± 0.56 ^b^	8.41 ± 0.50 ^b^	12.74 ± 0.60 ^b^
As (50 mg/kg) + NOB (25 mg/kg each)	12.39 ± 0.56 ^c^	5.08 ± 0.19 ^c^	3.28 ± 0.20 ^c^	5.97 ± 0.37 ^c^
NOB (25 mg/kg)	15.94 ± 0.51 ^a^	2.94 ± 0.12 ^a^	1.60 ± 0.05 ^a^	3.28 ± 0.11 ^c^

Values having different superscripts in the same column are significantly different from other groups.

## 4. Discussion

NOB administration remarkably reduced the bioaccumulation of As in the liver tissues. Phytochemicals are progressively replacing traditional chelating agents in preventing arsenic-induced toxicity. Flavonoids can chelate metal ions in different ways. Our results support earlier studies [25] that revealed that flavonoids act as valuable metal chelators due to their antioxidant behaviors. NOB is a unique flavonoid that showed an influential metal chelating capability, which reduced the bioaccumulation of As in the liver. Our results affirm the findings of Kumar and Pandey [26], who reported the metal-chelating properties of flavonoids. NOB, like other flavonoids, can use the same pathway to chelate As ions from liver tissues; however, the actual metal-chelating mechanism of NOB is still unknown.

In most organisms, the endogenous antioxidant activity of enzymes acts as the main line of defense in protecting against free radicals [27]. Assessing antioxidant enzyme activities is also critical to observe injuries instigated by As in hepatic tissues. SOD and CAT enzymes protect the body from the harmful effects of H_2_O_2_ lipid peroxidation prompted by oxidative stress [28]. SOD safeguards cells from ROS by converting superoxide into H_2_O_2_. Catalase and glutathione peroxidase are involved in the degeneration of H_2_O_2_ into non-toxic forms, such as H_2_O and O_2_, thereby eliminating the harmful effects of OH ions on the cells [29]. GSH fights against free radicals to protect membranes, which are essential in regulating tissue antioxidant defense systems [30]. The prolonged overproduction of ROS decreases the activity of these enzymes. It initiates oxidative injuries in hepatocytes, disrupting biological substances, such as DNA, proteins, and lipids [31]. Antioxidant enzyme activities and TAS levels were assessed to ascertain the antioxidant activity of NOB in the present study. It is reported that the level of TBARS validates liver injury. In this study, we noticed a noteworthy decrease in antioxidant enzyme activities and TAS levels, while TOS was elevated due to oxidative stress induced by As administration. Several studies have reported that the TOS level in testicular tissue increased in contaminant-exposed groups [32]. In comparison, NOB administration increased antioxidant ability and elevated TAS in liver tissues. It has been reported that NOB protects cells against hydrogen-peroxide-prompted damage by decreasing malonaldehyde (MDA), scavenging ROS, and increasing GSH and SOD contents [11]. It may be due to the healing ability of the NOB, which decreased TOS and ultimately increased TAS in the liver tissues. Our results were in line with the study by Jahan et al. who reported that NOB restored the oxidative stress altered by sodium arsenate exposure in human iPSCs-derived hNPCs [33].

The other noteworthy manifestation of As poisoning is an augmented concentration of H_2_O_2_ in the hepatic tissues of rats. The elevated level of H_2_O_2_ is a sign of decreased antioxidant profile and increased lipid peroxidation (LPO) in the hepatocytes of the rat. The fall in the antioxidant index and high oxidative stress production subsequently enhances the deposition of toxic As metabolites, which finally intensifies liver ailments [34]. Although, co-administration with NOB attenuated the oxidative trauma by inhibiting the oxidative stress that causes the recovery of antioxidant enzyme activity. Hepatotoxicity followed by As exposure leads to a considerable increase in TBARS. Increased free oxygen radical production initiates LPO, which has been accepted to be the most common cause of increased TBARS levels and is further associated with the decreased GSH level [35]. Thus, the depletion of GSH level increases As deposition in the liver, producing oxidative stress [36]. Recuperating TBARS and GSH content by cotreatment of NOB in our research work exhibited therapeutic potency against As-stimulated liver ailments in the rat.

Liver functioning tests are extensively used to measure liver conditions. Divergence in the measured results of serum markers (ALT, AST, and ALP) within the normal range can help diagnosis [37]. Aminotransferases such as ALT, ALP, and AST are enzymes that are an integral part of liver parenchyma cells. When the hepatic plasma membrane becomes injured, the enzymes that are present in the cytosol escape and move into the blood. As a result, these enzyme levels are sensitive indicators for detecting liver disorders [38]. Augmentation in serum biomarkers (ALT, AST, and ALP) in As-treated rats verified that the structure of hepatic tissues had been damaged as enzymes ordinarily present in the cytosol were released into the blood. Earlier research has shown that an increase in hepatic serum biomarkers implies greater oxidative damage to liver tissues, which is linked to hepatic impairment [39]. In comparison, cotreatment of NOB + As reduced the As-instigated liver damage by lowering the level of hepatic serum markers. 

Arsenic (AS) administration induced a profound increase in inflammatory markers levels, such as NF-κB, TNF-α, IL-1β, IL-6, and COX-2 activity. As evident, NF-κB is an inflammatory marker that quickly stimulates after internal or external stimulation in the cell, eventually augmenting the expression of TNF-α [40,41], IL-1β [42], IL-6 [40], as well as COX-2 activity [42]. Therefore, blocking the stimulation of NF-κB may implement a recurring role in reducing inflammation in a cell. However, in the present study, cotreatment of NOB lowered the level of the inflammatory marker, which might be ascribed to its anti-inflammatory potential.

Apoptosis is referred to as a programed cell death mechanism [43], which is categorized into intrinsic and extrinsic pathways [44]. Our investigation explored the innate path by estimating the expression of Bcl-2, Bax, Caspase-3, and Caspase-9. A class of proteins belonging to the Bcl-2 family maintains all the factors in this pathway. The protein family is categorized into pro-apoptotic (Bax) and anti-apoptotic (Bcl-2) proteins. Bax promotes the release of cytochrome c into the cytosol through the mitochondrial inter-membrane permeability, whereas Bcl-2 suppresses the release of cytochrome c [45]. A decrease in Bcl-2 and an escalation in Bax eventually changed the permeability of the mitochondrial inter-membrane, increasing the release of cytochrome c inside the cytosol [46]. The increased cytochrome c in cytosol ultimately triggers the activation of caspase-9, directing downstream caspase-3 activation to initiate cell apoptosis [47]. Caspase-3 performs a vital role in apoptotic pathways, and it is considered a reliable indicator for apoptosis [48]. The results of our investigation revealed that arsenic administration depleted the expression of Bcl-2, while escalating the expression of Bax, Caspase-3, and Caspase-9. Co-administration of NOB lowered the expression of Bax, Caspase-3, and Caspase-9, whereas it restored Bcl-2 gene expression. Our findings are further confirmed by the study of Li et al., who reported the antiapoptotic effect of NOB in the liver [49] and supported the assumption that the controlling ratio of Bcl-2/Bax might mediate the NOB protective effects against apoptosis.

The liver is considered a target organ for As exposure [50]. In the present study, liver histopathological manifestations were consistent with a biochemical evaluation that validated morphological variations in hepatocytes of arsenic-treated rats. As mediated hepatocytic degeneration and hepatic necrosis in rats [51]. Arsenic-induced toxicity increases LPO in the liver, which may lead to structural impairments. Pathological abrasions in the liver, such as focal inflammation, sinusoidal dilation, disordered liver lobules, congestion, central vein disruption, degenerated hepatocytes, hemorrhage, and necrosis, were seen in the As-intoxicated group. As deteriorates liver tissues due to raised lipid peroxides, linked with the biochemical alterations incited by As [52]. NOB treatment recovered the tissue damage toward normal histology of the liver. This could be attributed to the potent antioxidant potential of NOB. The chelation of As by NOB substantially decreased the oxidative threat, which may lead to the restoration of the histological architecture of the hepatic tissue.

## 5. Conclusions

Our results revealed that treatment with NOB in arsenic (As)-infected rats abated oxidative liver disruption generated by As. The administration of NOB substantially decreased the variations caused by As and attenuated oxidative trauma and hepatotoxicity. The restoration of serum enzyme levels raised the antioxidant defense profile and diminished inflammation, apoptosis, TBARS, and H_2_O_2_ concentrations. The hepatoprotective efficacy of NOB against As was further reinforced by the recovery in the histopathological anomalies prompted by As. In light of these findings, it can be deduced that NOB acts as an antioxidant agent which involves free-radical inhibiting and metal chelator, and, thus, amended the unfavorable state of hepatocytes, which unraveled its usage as a mitigator/attenuating agent in As-triggered hepatotoxicity.

## Data Availability

All the data have been presented in the manuscript.
